# Dynamics of Regulatory Networks in Gastrin-Treated Adenocarcinoma Cells

**DOI:** 10.1371/journal.pone.0078349

**Published:** 2014-01-08

**Authors:** Naresh Doni Jayavelu, Nadav Bar

**Affiliations:** Department of Chemical Engineering, Norwegian University of Science and Technology, Trondheim, Norway; Semmelweis University, Hungary

## Abstract

Understanding gene transcription regulatory networks is critical to deciphering the molecular mechanisms of different cellular states. Most studies focus on static transcriptional networks. In the current study, we used the gastrin-regulated system as a model to understand the dynamics of transcriptional networks composed of transcription factors (TFs) and target genes (TGs). The hormone gastrin activates and stimulates signaling pathways leading to various cellular states through transcriptional programs. Dysregulation of gastrin can result in cancerous tumors, for example. However, the regulatory networks involving gastrin are highly complex, and the roles of most of the components of these networks are unknown. We used time series microarray data of AR42J adenocarcinoma cells treated with gastrin combined with static TF-TG relationships integrated from different sources, and we reconstructed the dynamic activities of TFs using network component analysis (NCA). Based on the peak expression of TGs and activity of TFs, we created active sub-networks at four time ranges after gastrin treatment, namely immediate-early (IE), mid-early (ME), mid-late (ML) and very late (VL). Network analysis revealed that the active sub-networks were topologically different at the early and late time ranges. Gene ontology analysis unveiled that each active sub-network was highly enriched in a particular biological process. Interestingly, network motif patterns were also distinct between the sub-networks. This analysis can be applied to other time series microarray datasets, focusing on smaller sub-networks that are activated in a cascade, allowing better overview of the mechanisms involved at each time range.

## Introduction

Understanding gene transcription regulatory networks is critical to deciphering the molecular mechanisms resulting in different cellular states in response to growth factors [1], [2]. Gastrin is a peptide hormone that is mainly produced by G-cells in the stomach in response to a meal. It plays a key role in the physiological regulation of gastric acid secretion [3]. Gastrin binds to the cholecystokinin receptor-2 (CCKR-2), forming an active complex that initiates a signaling cascade [4]. The transduced signal results in different cellular processes such as growth, differentiation, proliferation, migration, angiogenesis and apoptosis [5]–[7]. Recent studies have revealed that gastrin can act as a co-risk factor for gastric carcinogenesis and atrophy in *Helicobacter pylori* infection [8], [9]. Dysregulation of gastrin can result in cancerous tumors, for example [10]. These cellular states are achieved through complex gene transcription regulation programs.

Gene regulatory networks are highly complex and dynamic, especially the coordinated regulation between transcription factors (TFs) and their target genes (TGs) [11]–[13]. At present, reconstructing these dynamic networks is a challenging task because only static TF-TG interaction data are available. In the gene regulation process, an active TF binds to the promoter region of a TG and initiates the process of transcription. The majority of transcription factors are not inherently active but become activated through complex mechanisms such as forming homo- or heterodimers, interacting with other signaling proteins and co-factors or binding to a specific microRNAs. The activity of a TF is dependent on the specific environment, cell type, and system dynamics. Thus, only specific TFs are active and regulate a set of TGs in a specific condition, resulting in a specific biological outcome in response to external stimuli. The group of active TFs and their regulated TGs are called active sub-networks or regulatory modules. For example, a regulatory module consisting of the TFs EGR4, FRA-1, FHL2 and DIPA promotes proliferation in breast cancer cells in response to epidermal growth factor (EGF) [2].

Understanding cellular functionality by studying its key components, interactions and network topological measures is a common approach in systems biology [14]–[17]. However, most of these studies are confined to static networks. Adding dynamic features to these studies may lead to new insights. Different active sub-networks have varying topological measures that characterize their biological functions. Thus, studying the active sub-networks in regulatory networks that respond to external signals may help to determine the cell fate.

It is well known that transcription networks often contain a small set of recurring regulatory patterns called network motifs. These small networks are frequently found in quantities that are significantly larger than would be expected for random networks [18]. These are the basic building blocks of any gene regulatory network and are found in the transcriptional networks of diverse organisms [19]. In the regulatory networks of *Saccharomyces cerevisiae* and *Escherichia coli*, a 3-node motif called a feed forward-loop and a four-node motif called a bi-fan are the most common [18]. These network motifs are unique for each active sub-network. Finding these motifs in active sub-networks can enhance our understanding of the design principles of complex regulatory networks.

In the current study, we combined time series gene expression data and static TF-TG interaction data to study the dynamic features of gastrin-regulated transcriptional networks. We reconstructed the dynamic activities of key TFs using network component analysis (NCA) [20], [21]. Our study identified four active sub-networks at four time ranges, namely immediate-early (IE), mid-early (ME), mid-late (ML) and very late (VL). These sub-networks showed variations in their network topological measures and network motif usage. In addition, gene ontology analysis of the TGs in each active sub-network demonstrated that each network is highly enriched with specific biological processes. Overall, our study provides a framework for studying dynamic transcription networks and obtaining new biological insights on the manner networks at specific time ranges as a response to external stimuli.

## Materials and Methods

### Data preprocessing

The gene expression data used in this study were obtained by measuring the response of AR42J adenocarcinoma cells treated with gastrin hormone at 11 time points over a period of 14 hours. We downloaded the data from GEO database (Array Express accession number: GSE32869) [22]. We applied loess normalization within time points and quantile normalization across time points [23]. The expression values were averaged over two replicate measurements. We conducted t-tests to identify differentially expressed genes (DEGs). The DEGs with p-value<0.05 at more than two time points were selected for further analysis [23]. This analysis resulted in 4105 DEGs. To reduce the noise and to smooth the data, we used Fourier transform functions to fit the time-series data. All the computations were performed using bioinformatics toolbox in MATLAB.

### Static regulatory network construction

We collected the experimentally verified TF-TG regulations from TFacts [24], a database containing experimentally validated regulations between 2720 TGs and 330 TFs. This database includes information integrated from different resources as TRED, TRDD, PAZAR NFIregulomeDB and their own experimental predictions. In addition we retrieved TF-TG interactions based on Chip-X experiments from Transcriptome Browser [25]. This list includes interactions between 312 TFs and 13133 TGs. Furthermore, we collected protein-protein interactions between TFs from BIOGRID [26] and HPRD [27] data bases. We integrated all the data to construct a static regulatory network of 449 TFs, 13398 TGs and 164077 unique interactions among them.

Active sub-networks for four time ranges were excerpted from static network by incorporating gene expression data and predicted TF activities from NCA. For each time range, active TFs and TGs (which displays higher expression/activity than a threshold) were identified and combined it with static network to define an active sub-network.

### Network component analysis (NCA)

Network component analysis (NCA) is a computational method for reconstructing hidden regulatory signals (TFs activity) from gene expression data with known connectivity information in terms of matrix decomposition [20], [28]. The NCA method can be represented in matrix form as follows:
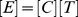
(1)where the matrix [*E*] represents the expression values of genes at various time points, the matrix [*C*] is the control strength of each TF on a target gene (TG), and the matrix [*T*] represents the activities of all of the TFs. The dimensions of [*E*], [*C*] and [*T*] are N X M (N is the number of targets, and M is the number of time points or measurement conditions), N X L (L is the number of TFs) and L X M, respectively.

Based on above formulation, the decomposition of [*E*] into [*C*] and [*T*] can be achieved by minimizing the following objective function:
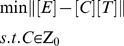
(2)where *Z*_0_ is the initial connectivity pattern. [*C*] and [*T*] are estimated using a two-step least-squares algorithm and are normalized through a nonsingular matrix [*S*] according to
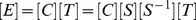
(3)


To guarantee the uniqueness of the solution for equation (3) up to a scaling factor, certain criteria, termed NCA criteria, must be satisfied:

- The connectivity matrix [C] must have full-column rank
- When a node in the regulatory layer is removed along with all of the output nodes connected to it, the resulting network must be characterized by a connectivity matrix that still has full-column rank
- The [T] matrix must have full row rank

Using NCA as the reconstruction method, we predicted significant TFs and their temporal activity profiles.

### Network topological metrics

The degree of a node *n*, *k(n)*, is the number of edges connected to it. The in-degree and out-degree of a node *n* is the number of edges with *n* as their terminal and initial nodes, respectively. The clustering coefficient, *C_n_* of a node *n* with *k* neighbors is the ratio of the actual number of edges *E_n_* between the neighbors to all the possible edges.
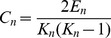
(4)


In the case of directed networks,
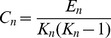
(5)


The betweenness centrality *C_b_(n)* of a node *n* is defined as follows.

In the case of directed networks,
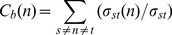
(6)where *s* and *t* are nodes in a network different from *n*, ***σ*** *_st_* denotes the number of shortest paths from *s* to *t*, and ***σ*** *_st_(n)* is the number of shortest paths from *s* to *t* that *n* presents in between. The closeness centrality *C_c_(n)* of a node *n* is defined as the reciprocal of the average shortest path length.
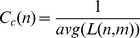
(7)where *L(n,m)* is the length of the shortest path between two nodes *n* and *m*. The mean values of all the topological metrics of all nodes in a network was computed to create a global view of the network. All computations are performed using the Cytoscape software tool [29].

### Network motif detection

Network motifs are small networks that are present in large complex networks at higher frequencies than in random networks. To understand the regulation patterns, we used the FANMOD tool [30], [31] to find 3- and 4-node sized network motifs. Significant motifs were identified based on a statistical measure, the Z-score. The Z-score of a network motif is defined as the difference between the frequencies in the original and random networks divided by the standard deviation.

### Gene ontology analysis

Gene ontology (GO) analysis aims to capture increasing knowledge of gene functions in a collective manner. We used the ClueGO tool [32] to find the highly enriched biological processes in active sub-networks. To calculate the enrichment values, we used a two-sided (enrichment/depletion) hypergeometric test, and p-values were adjusted for multiple testing using the Bonferroni method. ClueGO employs a new kappa static measure (ranging from 0 to 1) to link the terms or groups in the network. We chose a kappa score of 0.3. The size of the node in the network reflects the enrichment of the terms.

## Results and Discussion

**Figure 1:**
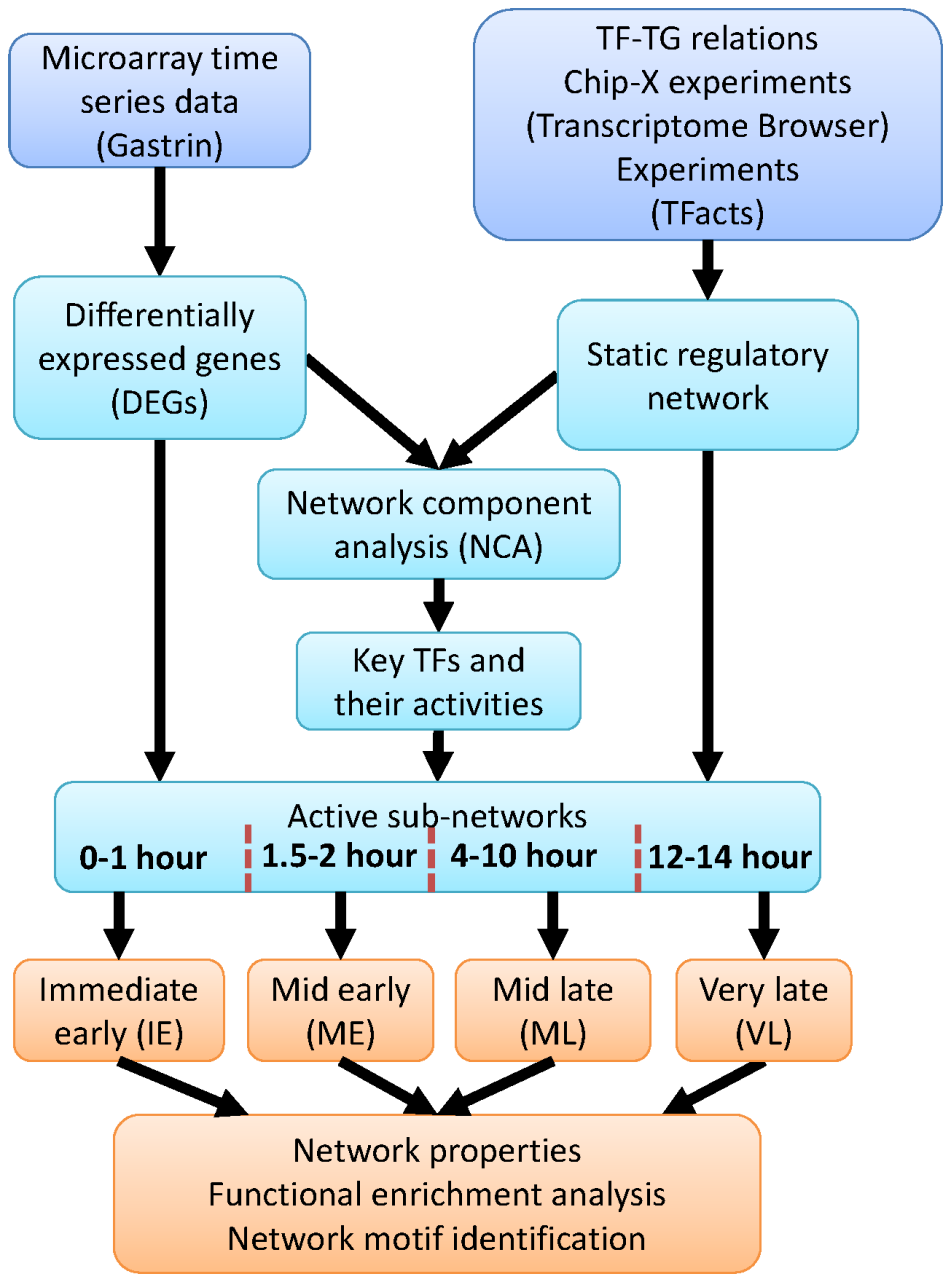
Schematic of the approach. The schematic of our approach for constructing the active sub-networks at different time ranges from gene expression data.

The workflow of our data pre-processing and subsequent analysis is presented in **Figure 1**. For this study, we used gene expression time series data of AR42J adenocarcinoma cells in response to gastrin measured at 11 time points over a period of 14 hours. The expression data provides the basis for our analysis of transcriptional regulatory network dynamics.

### Dynamics of gene expression and transcription factor activity

**Figure 2:**
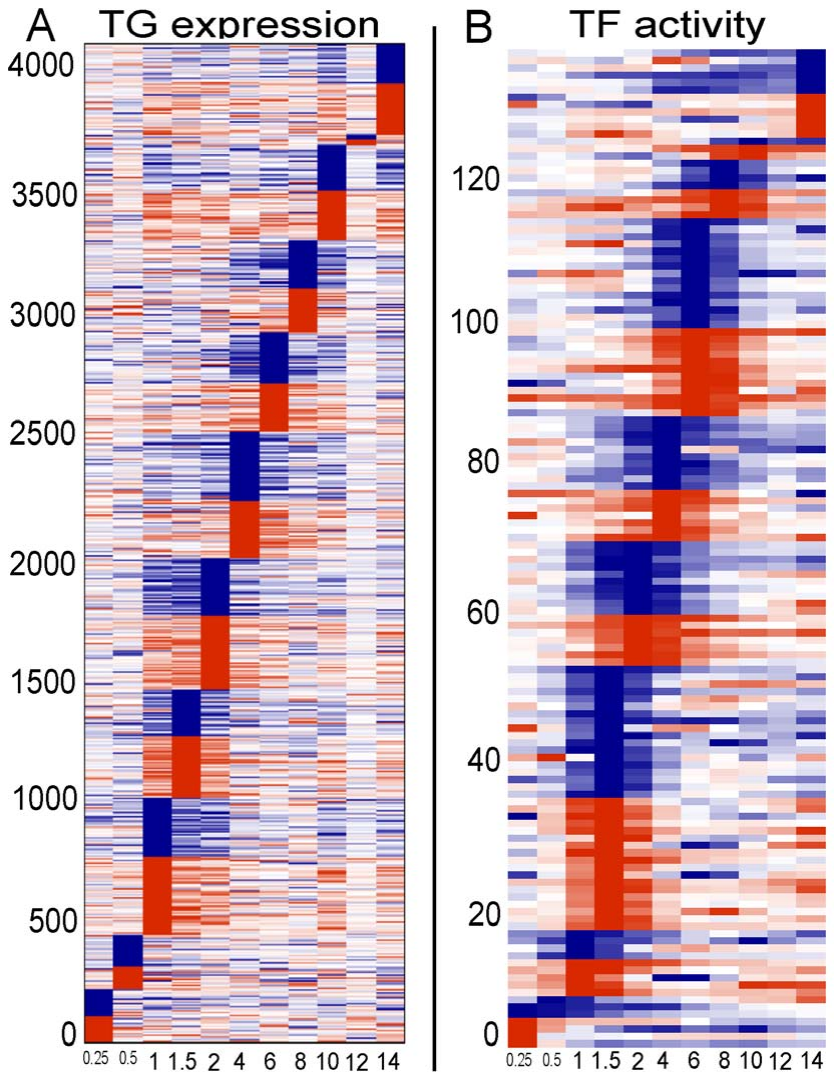
Dynamics of transcriptional regulation. (A) TG expression profiles. (B) Reconstructed TF activity profiles. Clustering was performed in such a way that both sequential and co-regulation was observed. Here, each row represents either a TG or TF, and each column represents a progression in time (in hours). Activations and repressions are represented by red and blue colors, respectively.

The changes in the expression of differentially expressed genes (DEGs) over the 14-hour period after gastrin stimulation are shown in **Figure 2A**. The gene expression data are clustered in a way such that we can observe both sequential and combinatorial regulation patterns. This expression pattern clearly displayed early-, mid-, and late-phase responses to gastrin stimulation. In addition, this expression pattern characterizes a typical stimuli response. The majority of the genes showed impulse response (exhibiting peak expression only at one time point), one of the typical response patterns in time series data [33].

We used network component analysis (NCA) [20] to reconstruct the dynamic activities of TFs from gene expression data and a known TF-TG topology (see Methods section). The computed TF activities were also clustered in a pattern similar to that of the gene expression shown in **Figure 2B**. This model included a number of TFs, such as ATF2, CREB1, ELK1, EGR1, FOXO1, and SP1, which are known to be regulated by the gastrin-cholecystokinin receptor-2 (CCKR-2) signaling pathway [34]–[39].

**Figure 3:**
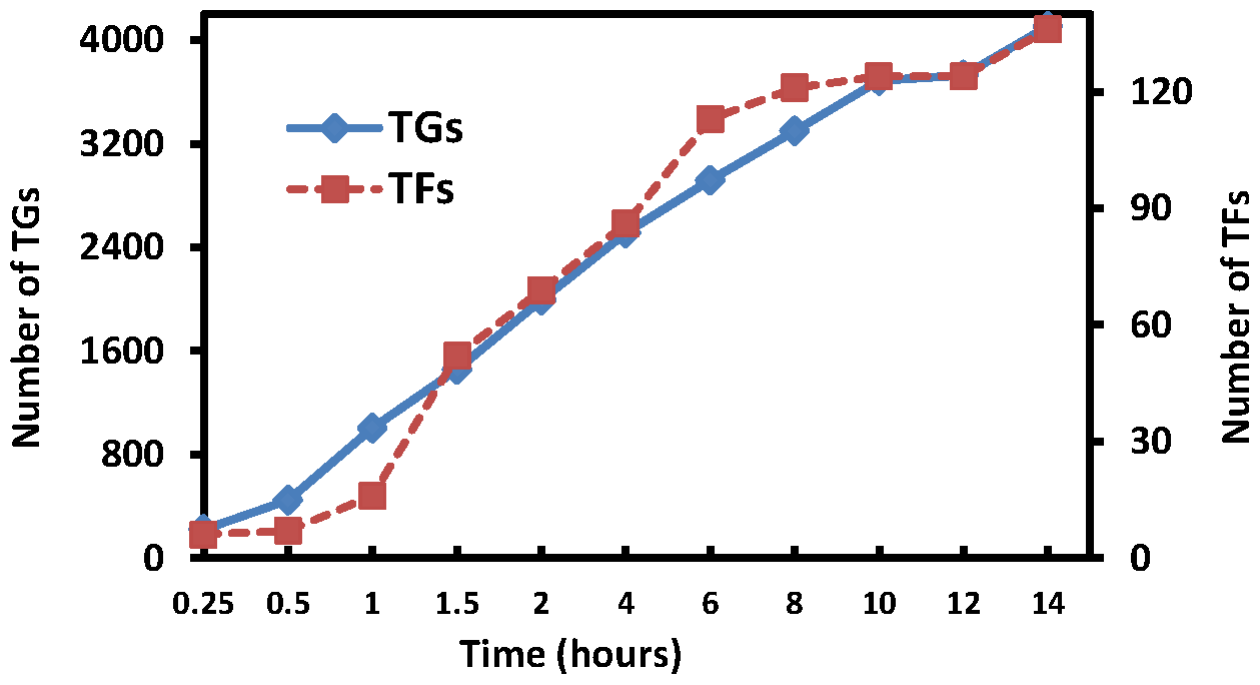
Differential regulation. The number of active TGs and TFs at each time point is presented. Active TGs and TFs were defined at each time point based on peak expression or activity at that time point.

To understand gastrin regulation in adenocarcinoma cells in terms of quantitative measurements, we computed the number of differentially expressed genes (DEGs) and TFs (computed from NCA) activated at each time point (**Figure 3**). It appears that during the initial time points, TGs expressions were activated by gastrin intracellular signaling which in turn activated a large number of TFs at later time points (1.5 hours after gastrin stimulation). The largest effects of gastrin on the transcriptome were found at 1 and 2 hours for TGs and 1.5 and 6 hours for TFs. At 2 hours, ∼13% of TGs were differentially expressed and, interestingly, ∼12.5% of TFs were active at the same time. Surprisingly, none of the TFs were showed peak activity at 12 hours, and only TGs were also only 1% showed peak expression.

### Active sub-networks

To study the dynamic features of regulatory networks, a static integrated regulatory network was constructed by combining various data resources including Chip-X studies, experiments and predicted data bases. The regulatory TF-TG relationships were extracted from the Transcriptome Browser [25] and TFacts [24]. The interactions between TFs were retrieved from the BIOGRID and HPRD databases [26], [27]. The combined integrated network contained 449 TFs, 13398 TGs and 164077 unique interactions among them (see **File S1**).

The active sub-networks were built based on differential expression of TGs and peak TF activity during a specific time range. We defined four active sub-networks, namely immediate-early (IE), mid-early (ML), mid-late (ML) and very late (VL), as shown in **Figure 1**. First, we identified active TGs and TFs (showing expression or activity higher than a specified threshold) at each time point. We retrieved interactions between active TGs and TFs from a static regulatory network (see **File S2**).

We then examined the structural and modular architecture of the four constructed active sub-networks. This analysis clearly distinguished each active sub-network from the others. The size and topological metrics of the static regulatory network and the four active sub-networks are presented in **Table 1**. The size of the VL active sub-network was significantly smaller than the others. The clustering coefficient (CC), which defines the interconnectivity of nodes in the network, was higher in the ML and VL sub-networks. This indicates that the ML and VL active sub-networks were less organized in terms of modular structures than the IE and ME networks. The values for the average path length (APL) and betweenness centrality (BC) were greater in the ME and VL active sub-networks respectively than compared to the other sub-networks. The higher BC in the VL network and APL in the ME sub-networks suggests that they are controlled by a number of central nodes. Closeness centrality was higher in the ME and VL networks than IE and ML sub-networks. This suggests that ME and VL networks have more nodes with shortest paths to other nodes.

**Table 1:** Topological properties of networks.

Network		Static network	IE network	ME network	ML Network	VL network
	Active TGs	12980	734	877	1201	265
Size	Active TFs	449	43	78	87	31
	Regulatory interactions	164077	2008	3957	5984	762
	Average degree	24.03	5.02	8.02	8.94	4.82
Topological metrics	Clustering coefficient	0.274	0.054	0.062	0.113	0.272
	Diameter	6	7	9	7	6
	Average path length	2.634	2.957	3.343	2.942	2.697
	Betweenness centrality	3.87E-06	7.48E-05	15.92E-05	9.07E-05	42.52E-05
	Closeness centrality	1.38E-02	2.94E-02	3.39E-02	2.71E-02	5.64E-02
	Centralization	0.285	0.338	0.245	0.293	0.456
	Average degree	NA	1.26±0.41	1.76±0.61	2.45±0.66	0.61±0.32
	Clustering coefficient	NA	0.029±0.023	0.05±0.03	0.077±0.04	0.014±0.02
	Diameter	NA	6.01±1.31	7.1±1.63	7.6±1.24	2.9±1.27
	Average path length	NA	2.66±0.57	2.99±0.5	3.23±0.37	1.52±0.46
	Centralization	NA	0.06	0.151±0.05	0.181±0.06	0.109±0.06

The degree of a node is the number of interactions incident to it. The clustering coefficient measures the interconnectivity around a node. The average path length is the average length of all shortest paths among all node pairs. Betweenness centrality is the average number of shortest paths between all node pairs passing through a node. Closeness centrality is the reciprocal of the average shortest path lengths. The mean and standard deviation (mean ± SD) of 100 random networks for each active sub-network are presented in the last row. All computations were performed in Cytoscape.

NA-Denotes not applicable.

To corroborate the significance of these topological divergences, we performed the same computations on a set of random networks created from a static network with the same number of nodes as the actual active sub-networks. We generated 100 random networks, and the structural properties were averaged over these 100 networks. The mean and standard deviations are provided in the last row of **Table 1**. All the structural properties of actual sub-networks were significantly different from those of random networks except for network diameter, indicating that the computed network properties are indeed biologically significant. We performed all these computations using the Cytoscape software tool [29].

### Network motifs

Transcriptional regulatory networks are made up of small recurring patterns called network motifs. To understand the dynamic functional characteristics of the gastrin network, we performed a network motif analysis for each active sub-network. We identified network motifs with 3-, 4- and 5-nodes in four active sub-networks using the FANMOD tool [30], [31]. The statistical significance of a network motif is computed by comparing occurrences of the motif in an active sub-network and in random networks. The Z-score of a motif is determined as the difference between its occurrence in an active sub-network and in hundreds of random networks with a normalized standard deviation.

**Figure 4:**
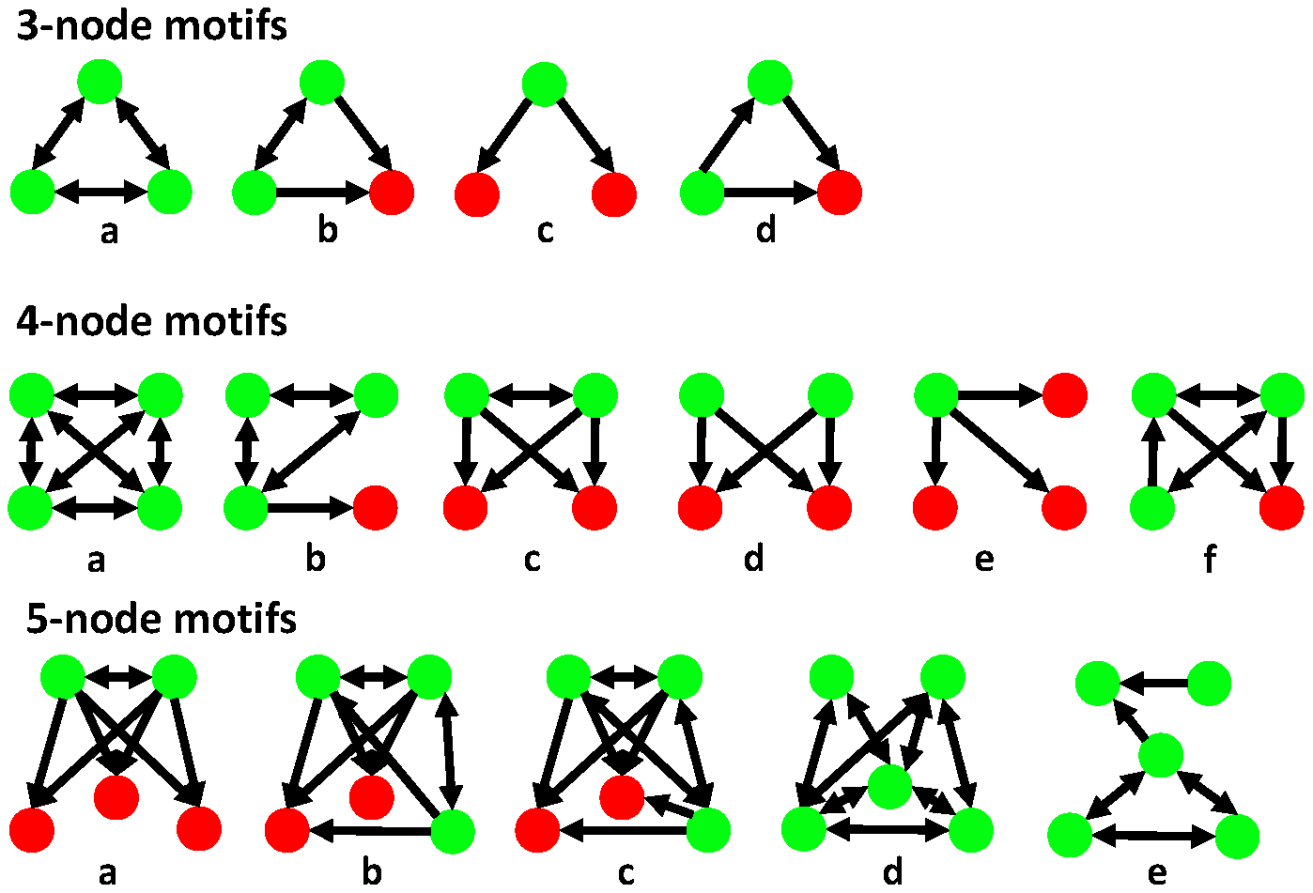
Network motifs. The key network motifs of 3-, 4-, and 5-component nodes detected in four active sub-networks are presented. Red nodes represent TGs and green nodes represent TFs. The network motif search was performed using the FANMOD tool. The motifs with p-value<0.05 were considered statistically significant.

**Figure 5:**
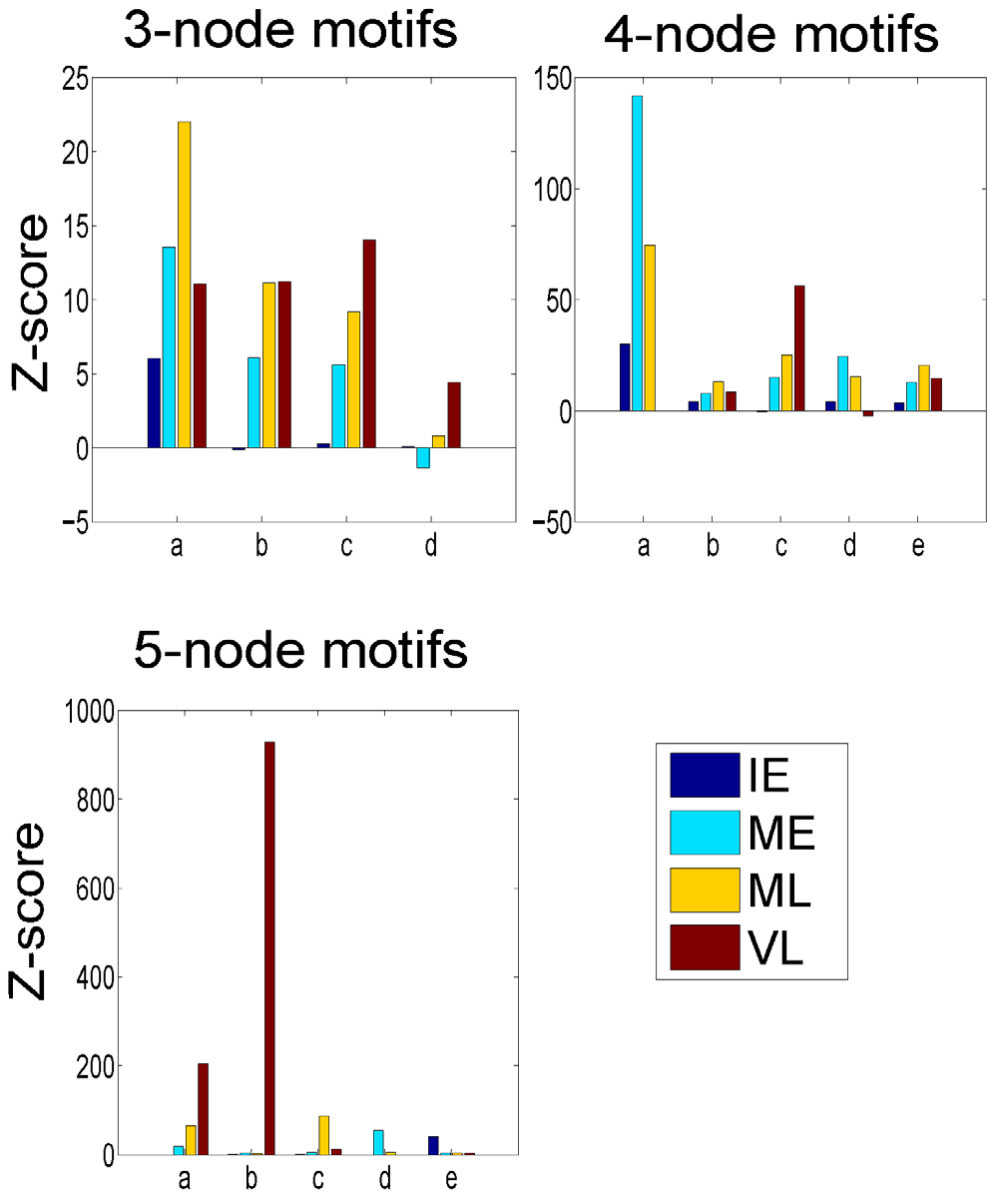
Differential usage of motifs. The computed Z-scores for selected network motifs across the four active sub-networks were shown. Each active sub-network is enriched in specific network motifs. The labels ‘a’, ‘b’, ‘c’, ‘d’, and ‘e’ represent the respective network motifs in **Figure 4**. The network motif search was performed using the FANMOD tool. The motifs with p-value<0.05 were considered statistically significant.

The significantly enriched network motifs and their Z-scores in the four active sub-networks are presented in **Figures 4** and **5**, respectively (see **Files S3 to S10** for complete results). We found four variants of 3-node motifs that were overrepresented in the four active sub-networks. Cliques (three TFs regulating each other, type ‘a’), cross-regulating TFs co-targeting a TG (type ‘b’), SIMs (single input motifs, type ‘c’), and FFLs (feed forward loops, type ‘d’) are the various types of significantly enriched 3-node motifs. Cliques were most frequently used motif pattern in all active sub-networks, suggesting that formations of three interacting TFs regulatory complexes are involved during the whole response. Network motif types ‘b’ and ‘c’ were significantly missing in the IE network, suggesting that they are not main cause for the early activation of large number of TGs. Cliques and cross-regulating TFs co-targeting a TG (type ‘b’) were previously found in the integrated regulatory networks of *Saccharomyces cerevisiae* [40]. FFLs are well-known regulatory patterns in transcriptional networks [14], [15], [19], [41]. Six variants of 4-node motifs appeared more frequently than others. Interestingly, cliques of four TFs were not found in the VL active sub-network. Two variants of bi-fan motifs (types ‘c’ and ‘d’) were found. Bi-fan motifs type ‘c’ and ‘d’ were enriched in the VL network and ME sub-networks, respectively. Bi-fan motifs are well-known regulation patterns in gene regulatory networks [18], [40], [42]. Another commonly identified network motif was SIM (single input motif, type ‘e’), which was detected more prominently in all the sub-networks except in the IE sub-network. SIMs play a key role in large-scale gene activation [15], [19], [43].

Five types of 5-node motifs were enriched in the constructed networks. MIMs (multi input motifs, types ‘a’, ‘b’ and ‘c’) were the most common regulatory pattern among 5-node motifs. Of these, type ‘b’ was significantly enriched in the VL sub-network and type ‘c’ in the ML network. MIMs are also known to regulate large-scale gene activation [15], [43]. It is possible that these motifs are also responsible to turn off expression of large number of genes, since here they mostly appear at late time ranges. Additionally, the IE and ME active sub-networks were uniquely enriched with types ‘e’ and ‘d’, respectively, and these network motifs were not found in other sub-networks. This may suggest that large TF regulatory complexes are responsible for large scale gene expression and as an immediate response to stimuli. The current literature on 5-node motifs is very limited [44]. Thus, network motif analysis disclosed basic regulatory patterns in the four active sub-networks, and each sub-network was enriched with different network motifs. Although the findings show differential usage of motifs by each active sub-network, these results should be confirmed experimentally.

### Functional annotation of differentially expressed genes

**Figure 6:**
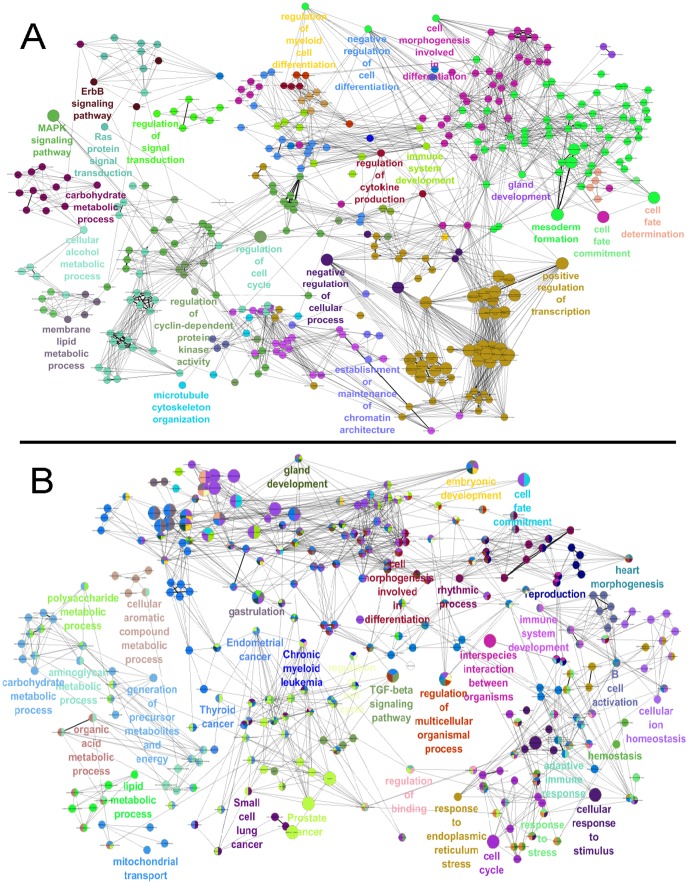
Functional enrichment analysis. Network representations of enriched terms among active genes in the respective sub-networks. Enriched terms are represented as nodes based on their kappa score (≥0.3). The node size indicates the significance of the enrichment. (A) IE active sub-network. (B) ME active sub-network.

To identify which and how various biological processes are affected by differentially expressed TGs in each active sub-network, we conducted functional annotations in ClueGO tool in Cytoscape [32]. All statistically significant functional terms in the interacting network for the four active sub-networks are presented in **Figures 6** **and** **7**. Interestingly, several unique terms for each active sub-network and few common terms among all sub-networks were found.

**Figure 7:**
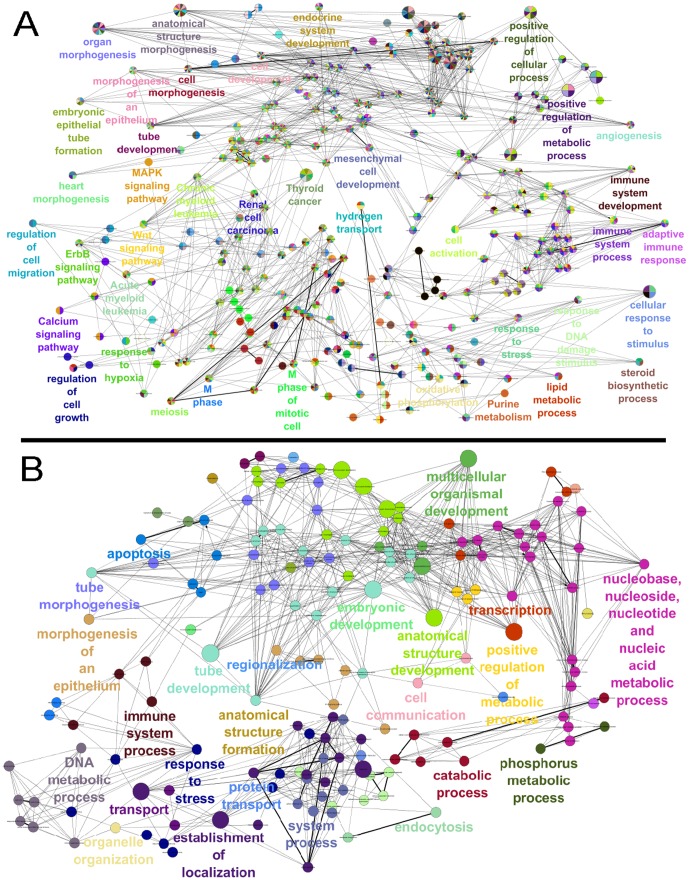
Functional enrichment analysis. Network representations of enriched terms among active genes in the respective sub-networks. Enriched terms are represented as nodes based on their kappa score (≥0.3). The node size indicates the significance of the terms enrichment. (A) ML active sub-network. (B) VL active sub-network.

The IE active sub-network is highly enriched in functions involved in cellular differentiation. Many previous studies have confirmed the role of gastrin in differentiation processes [45]–[47]. Koh et al. demonstrated that gastrin deficiency in mouse models altered the differentiation of gastric mucosa [46]. Additionally, Wang et al. showed gastrin-induced differentiation of rat intestinal epithelial cells in correlation to the up regulation of villin expression [47]. The other significant categories are regulation of several signal transduction pathways as ErbB signaling pathway, MAPK signaling pathway, Ras protein signal transduction and cell fate determination, cell fate commitment. Several studies have shown that stimulation of gastrin-CCK2R resulted in the activation of various signal transduction pathways including MAP kinases [48]–[52]. Gastrin induced the proliferation of AR42J cells by activating MAPKs and the c-fos gene [53].

The ME active sub-network is enriched in a large number of significant categories involving several metabolic and several types of cancer associated pathways. This sub-network is enriched in energy-related metabolic processes such as carbohydrate, polysaccharide, lipid, organic acid, cellular aromatic compound metabolic processes and generation precursor metabolites and energy processes. This network is overrepresented by cancer pathways such as small cell lung cancer, thyroid cancer, prostate cancer, endometrial cancer and chronic myeloid leukemia. In addition, this network is enriched with response to endoplasmic reticulum (ER) stress, cellular response to stimulus, B cell activation and hemostasis. One of the recent studies confirms the involvement of gastrin in regulating the genes resulting in ER stress [54].

The ML network is highly enriched in biological processes related to morphogenesis such as the cell morphogenesis, morphogenesis of an epithelium, embryonic epithelial tube formation, heart morphogenesis. In addition, this network is enriched with regulation of cell migration and cell growth, angiogenesis and activation of Wnt and calcium signaling pathways. Previous studies of gastrin-CCK2R (cholecystokinin-2 receptor) signaling have confirmed some of the processes predicted in this study. Gastrin-CCK2R involvement in the morphogenesis of epithelium cells was found in previous studies. Pagliocca et al. found that stimulation with gastrin promotes branching morphogenesis (process of tubule formation) in gastric AGS cells through the activation of protein kinase C (PKC) [55]. Previous studies have shown the involvement of gastrin in angiogenesis of various types of cells [56], [57]. Lefranc et al. investigated the role of gastrin on angiogenesis in gliomas both in vitro and in vivo. This study concluded that gastrin has marked proangiogenic effects in human glioblastomas and endothelial cells [56].

The VL sub-network is mainly involved in metabolic processes such as DNA metabolic process, phosphorous metabolic process, cell communication, apoptosis and tube morphogenesis. Gastrin has induced apoptosis in many cells in previous studies [52], [58]. Muerkoster et al. performed an in vivo study to determine the role of gastrin in colon carcinogenesis and found that gastrin was able to induce apoptosis in human colon cancer cells through the wild-type CCK2 receptor, thereby suppressing the growth of colon cells [58].

Thus, our functional annotation of genes in four active sub-networks revealed several known and new functions of gastrin. In addition, this analysis contributed to identifying the gastrin response from a dynamic perspective.

## Conclusion

The primary objective of this study was to analyze the time series gene expression data generated by external stimuli to understand the transcriptional regulatory network from a dynamic perspective. To achieve this goal, we integrated information from a static TF-TG network with gene expression data to identify key TFs temporal dynamics. The gene expression and TF activities showed early-, mid-, and late-phase action in response to gastrin. This indicates that gastrin regulates genes over a period of 14 hours, although the majority of the genes were active at 1 and 2 hours and TFs were active 1.5 and 6 hours after gastrin treatment.

To more comprehensively understand the mechanisms of transcriptional regulation, we built four active sub-networks at four different time ranges. The active sub-networks defined in this study showed structural differences in their network organization. The ME and VL sub-networks were more strongly interconnected than the others. In addition, we identified key regulatory patterns, called network motifs, in all sub-networks. This analysis showed that distinct network motifs were significantly enriched in each active sub-network. The GO ontology and pathway analysis of active TGs and TFs in each active sub-network revealed interesting facts. Each active sub-network was enriched in unique GO terms/pathways. This shows that gastrin triggers different cellular states through diverse and complex transcription regulation patterns depending on the time of activation. We demonstrated that analyzing time series microarray data through partitioning to smaller temporal sub-networks reveals network properties that are unique for each time range, yet may otherwise be hidden when the whole time range is combined.

The development of high-throughput technologies such as microarrays results in large amounts of biological data and demands the rapid development of computational methods and strategies to analyze the data and thus extract biological knowledge. Our current study provides one such strategy for using these data and integrating known biological information to decipher the mechanisms of signaling and transcriptional programs of the biological system.

## Supporting Information

File S1 **Integrated static network.** The integrated static network from different sources used in the analysis of this study is provided as a Cytoscape network file.
(ZIP)

File S2 **Active sub-networks.** The four active sub-networks extracted at different time ranges in gastrin signaling are provided as a Cytoscape network file.
(ZIP)

File S3 **Network motif analysis.** The complete results of the 3-node network motif analysis for the IE active sub-network are provided as text file.
(OUT)

File S4 **Network motif analysis.** The complete results of the 3-node network motif analysis for the ME active sub-network are provided as text file.
(OUT)

File S5 **Network motif analysis.** The complete results of the 3-node network motif analysis for the ML active sub-network are provided as text file.
(OUT)

File S6 **Network motif analysis.** The complete results of the 3-node network motif analysis for the VL active sub-network are provided as text file.
(OUT)

File S7 **Network motif analysis.** The complete results of the 4-node network motif analysis for the IE active sub-network are provided as text file.
(OUT)

File S8 **Network motif analysis.** The complete results of the 4-node network motif analysis for the ME active sub-network are provided as text file.
(OUT)

File S9 **Network motif analysis.** The complete results of the 4-node network motif analysis for the ML active sub-network are provided as text file.
(OUT)

File S10 **Network motif analysis.** The complete results of the 4-node network motif analysis for the VL active sub-network are provided as text file.
(OUT)
